# Reactivation of Latent Varicella-Zoster Virus Leading to Meningitis in a Patient With Malignant Pleural Mesothelioma Undergoing Immune Checkpoint Inhibitor Therapy: A Case Report

**DOI:** 10.7759/cureus.88696

**Published:** 2025-07-24

**Authors:** Risako Shionoya, Shun Ishiyama, Hirohiko Ono, Chihiro Sasaki, Naoto Sugeno, Takuya Saito, Takashi Kikuchi, Tomohiro Ichikawa, Risa Ebina-Shibuya, Hisatoshi Sugiura

**Affiliations:** 1 Department of Respiratory Medicine, Tohoku University Graduate School of Medicine, Sendai, JPN; 2 Department of Neurology, Tohoku University Graduate School of Medicine, Sendai, JPN; 3 Department of Medical Science and Innovation, SiRIUS Institute of Medical Research, Tohoku University, Sendai, JPN

**Keywords:** immune checkpoint inhibitor, immunotherapy infections due to dysregulated immunity, iris, iti-di, meningitis, varicella zoster virus

## Abstract

Immune checkpoint inhibitor (ICI) therapy represents a significant advancement in cancer treatment through immune system activation. However, ICI presents paradoxical immunotherapy infections due to dysregulated immunity (ITI-DI). Here, we report varicella-zoster virus (VZV) meningitis in a patient with malignant pleural mesothelioma undergoing ICI therapy, characterized by disturbed consciousness and cerebrospinal fluid abnormalities. Although antiviral therapy with acyclovir effectively reduced VZV DNA to undetectable levels, persistent cerebrospinal fluid mononuclear pleocytosis was observed, which is akin to immune reconstitution inflammatory syndrome (IRIS). This case highlights the complex immunological interplay during ICI therapy, where immune activation paradoxically creates vulnerability to certain infections through mechanisms distinct from classic immunosuppression. Our findings underscore the importance of recognizing IRIS and ITI-DI as distinct entities requiring specific clinical consideration. We propose that comprehensive monitoring for atypical infections and implementation of pathogen-specific management strategies are essential components of care for patients receiving ICI therapy, particularly given the unique immunological milieu these treatments create.

## Introduction

Varicella-zoster virus (VZV) meningitis is severe and often fatal, requiring a comprehensive analysis of cerebrospinal fluid (CSF) for prompt diagnosis and treatment. Immune checkpoint inhibitor (ICI) therapy is a type of immunotherapy that aims to shrink tumors by reactivating cytotoxic T cells suppressed by tumor cells. Notably, the immune-related adverse event (irAE) encephalomeningitis in patients undergoing ICI therapy is clinically similar to VZV meningitis. While infectious complications during ICI therapy are generally rare, there have been occasional case reports of VZV infections [1,2].

In addition to classic immunosuppression-related infections, a new category termed immunotherapy infections due to dysregulated immunity (ITI-DI) [3] and the concept of reactivation of latent/chronic infection similar to immune reconstitution inflammatory syndrome (IRIS) [4,5] have been recognized. ITI-DI refers to infections that arise from a hyperinflammatory and dysregulated immune response triggered by ICI therapy, even in the absence of immunosuppressive medications. Immune checkpoints, such as programmed death 1 (PD-1) and cytotoxic T-lymphocyte-associated antigen 4 (CTLA-4), act as essential brakes on immune responses, helping to maintain immune balance and prevent excessive inflammation. Blockade of these checkpoints by ICIs can disrupt this delicate equilibrium, resulting in an overactive immune system that may lose tolerance to self-antigens and commensal or latent microorganisms, thereby increasing the risk of pathological immune activation [3,6]. By interfering with these regulatory pathways, ICIs can trigger the reactivation of latent infections, including tuberculosis and VZV. The hyperinflammatory environment that arises from uncontrolled T-cell activation not only exacerbates tissue damage but also sustains conditions that enable dormant pathogens to reactivate. As a result, the normal immune regulation required to contain chronic or latent infections is compromised, illustrating a paradox where immune enhancement may inadvertently precipitate serious infectious complications that are distinct from traditional immunosuppression, presenting a unique clinical challenge in patients receiving cancer immunotherapy. IRIS is a syndrome that occurs when the recovering immune system overreacts to infectious agents. Although ICI therapy reactivates cytotoxic T cells, which typically prevents VZV reactivation associated with declining cellular immunity, it may also induce immune recovery, potentially leading to the reactivation of latent/chronic infections, similar to IRIS.

Here, we report a case of VZV meningitis in a patient with malignant pleural mesothelioma undergoing ICI therapy, which may have developed in association with ITI-DI or IRIS. By detailing our case, this report illustrates how ITI-DI and IRIS must be considered in contemporary oncologic practice to better manage emerging immunotherapy-related complications and reduce morbidity and mortality in this growing population of patients treated with ICI therapy.

## Case presentation

**Figure 1:**
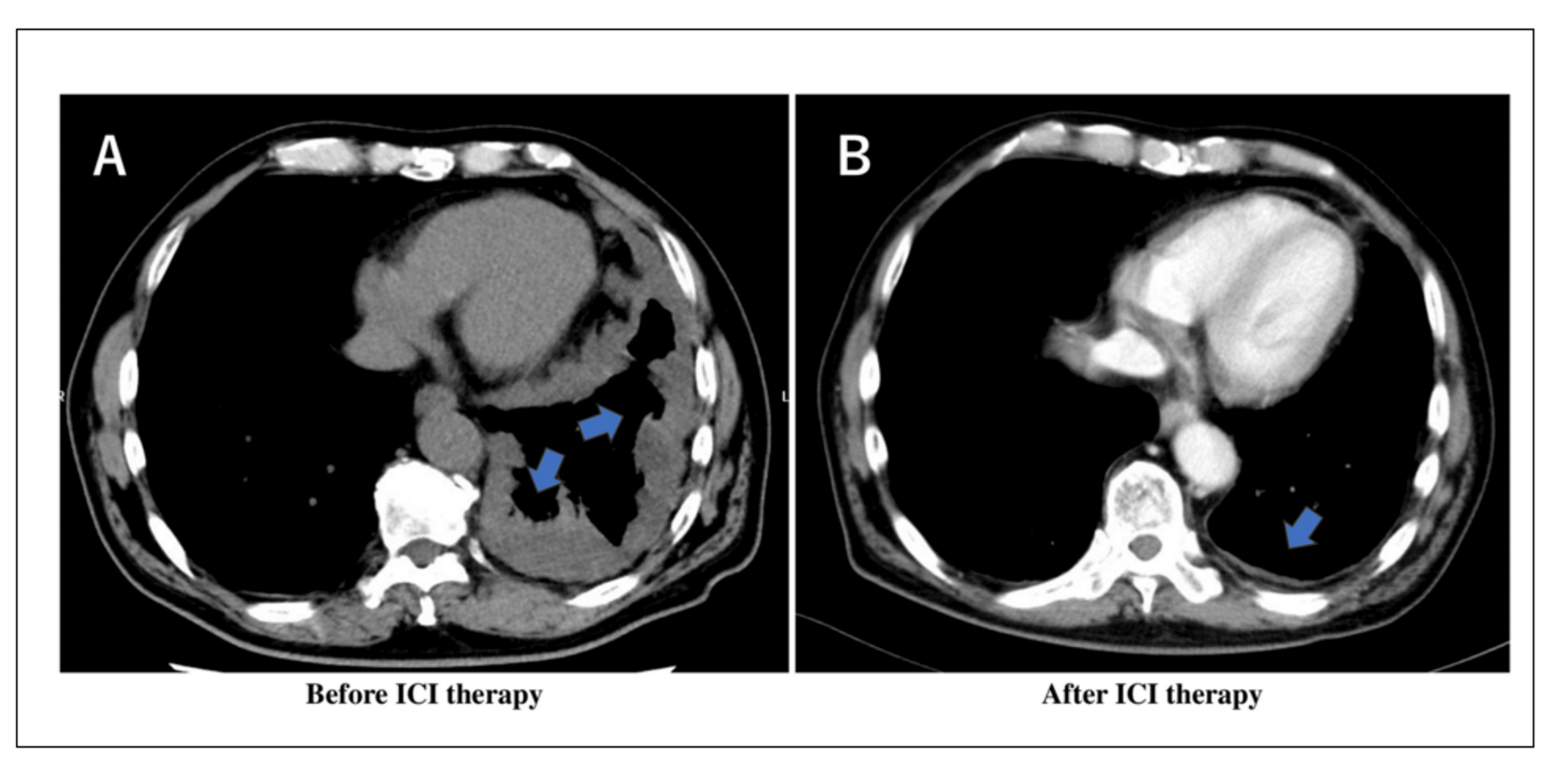
Chest CT image before and after ipilimumab and nivolumab combination therapy (A) Chest CT image before the initiation of ipilimumab and nivolumab combination therapy. (B) Chest CT image after three courses of ipilimumab and nivolumab combination therapy. ICI: Immune checkpoint inhibitor.

A 73-year-old male with epithelial malignant pleural mesothelioma presented with an acute onset of neurological symptoms after receiving three courses of ipilimumab and nivolumab combination therapy (Figure 1). He was not using steroids or immunosuppressive drugs and had no history of diabetes, human immunodeficiency virus (HIV), or apparent shingles. He had not received the shingles vaccination. Six days after his last nivolumab treatment, he developed disturbed consciousness, unstable gait, headache, and fever.

**Figure 2:**
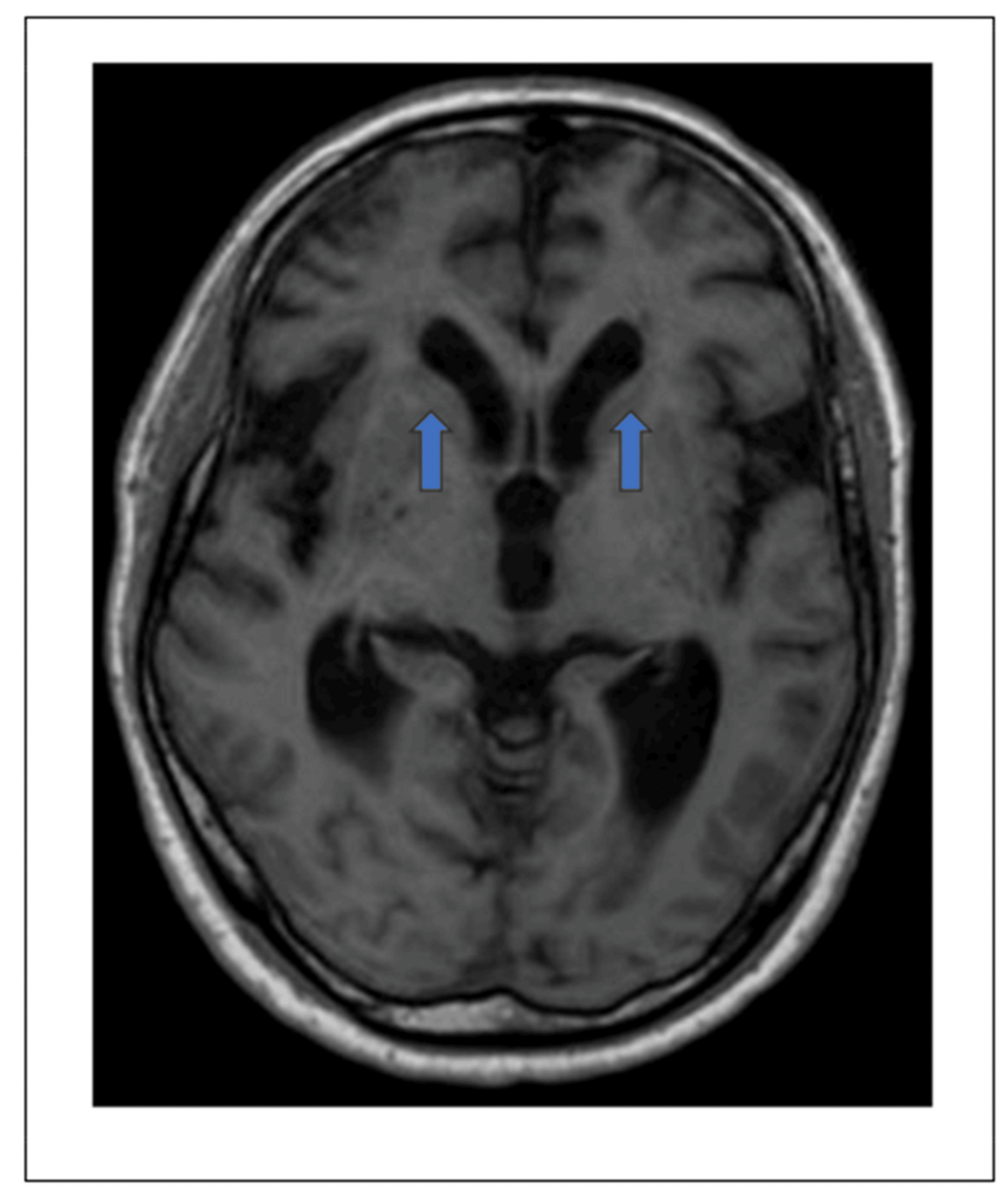
Cerebral magnetic resonance imaging

On admission, vital signs were as follows: Glasgow Coma Scale score of 9 (E3, V3, M3), a body temperature of 38.7℃, blood pressure of 211/125 mmHg, heart rate of 86 beats per minute, respiratory rate of 20 breaths per minute, and oxygen saturation of 95% while on room air. Physical examination revealed positive meningeal signs and Babinski signs. Notably, no skin rash was observed at this time. Cerebral magnetic resonance imaging (MRI) on admission showed slight ventricular dilatation without evidence of focal lesions, cerebral edema, or abnormal enhancement - findings that were nonspecific but did not suggest acute infarct, hemorrhage, or classical features of autoimmune encephalitis (Figure 2).

The initial differential diagnosis included both encephalitis as an irAE and infectious encephalitis of bacterial or viral origin. The fact that neurological symptoms developed soon after ICI therapy - without any rash present at onset - raised significant concern for an immune-mediated central nervous system (CNS) complication, highlighting the diagnostic challenge in distinguishing between these conditions. Lumbar puncture demonstrated an elevated opening pressure (>300 mmH_2_O), pleocytosis with mononuclear predominance, and increased protein levels. The CSF was positive for VZV DNA. These CSF findings - markedly elevated opening pressure, lymphocytic pleocytosis, elevated protein, normal or mildly decreased glucose, and positive VZV DNA - together confirmed the diagnosis of VZV meningitis.

**Figure 3:**
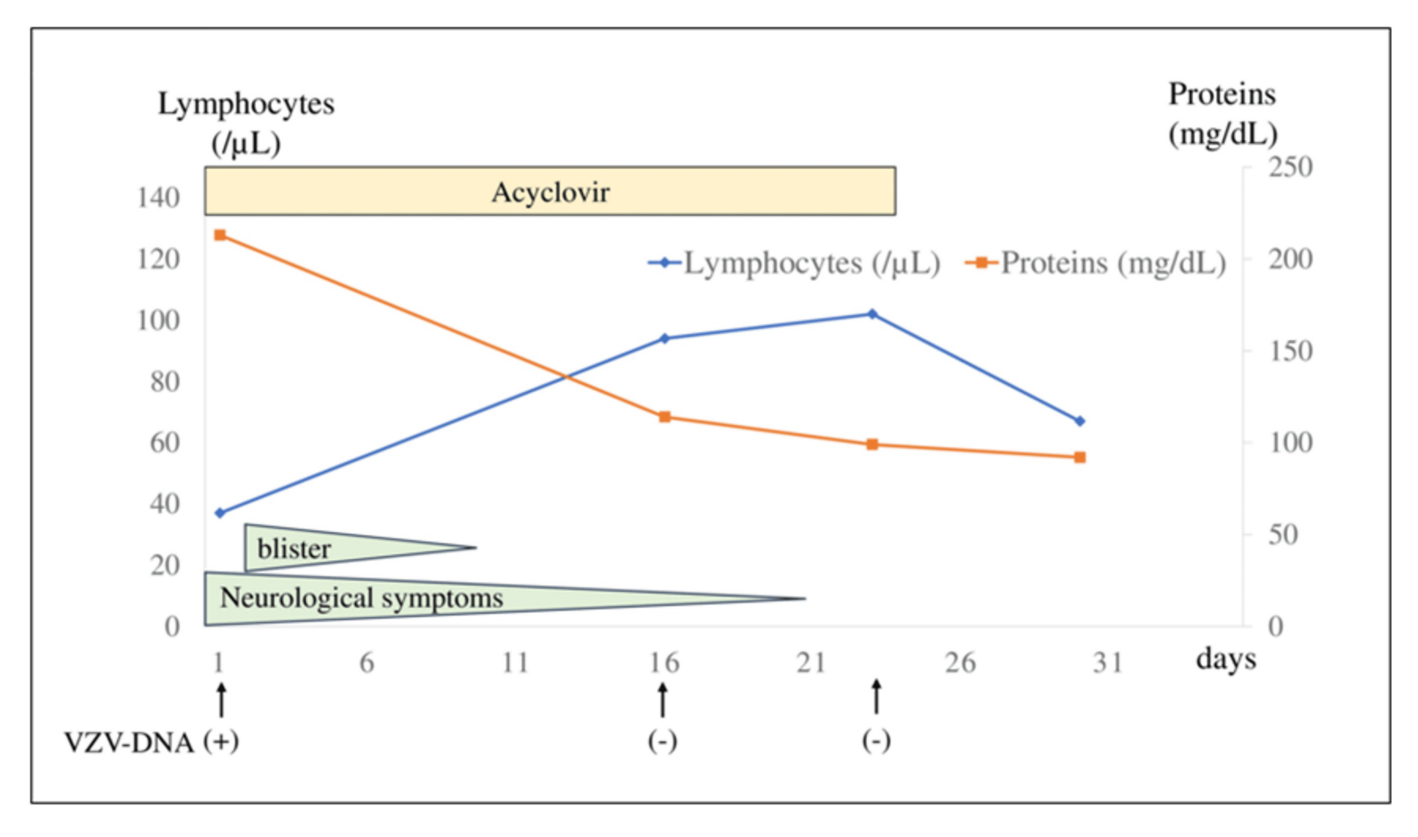
Clinical course and treatment of the patient The number of lymphocytes and protein levels in the cerebrospinal fluid, the result of the varicella-zoster virus (VZV) DNA test, and clinical symptoms are shown in this figure.

Intravenous acyclovir was initiated at 10 mg/kg/day (750 mg/day). On the second day of hospitalization, a typical herpes zoster eruption appeared at the mandibular region, neck, and right thigh. By the ninth day, the cutaneous lesions had crusted, and the patient demonstrated significant improvements in neurological symptoms. Due to concerns about potential false negatives for VZV DNA and possible immune complications, CSF analyses were repeated to monitor cell counts. Subsequent CSF analyses on days 16 and 23 were negative for VZV DNA, but pleocytosis persisted unexpectedly. Acyclovir therapy was continued for 23 days, ensuring two consecutive negative VZV DNA results. Despite ongoing CSF pleocytosis, cell counts decreased by day 30 following the cessation of antiviral therapy (Figure 3 and Table 1). Mesothelioma treatment was stopped after meningitis onset, but the patient has remained relapse-free and under observation for over one year.

**Table 1:** Total cell counts, lymphocyte counts, protein concentrations, and glucose concentrations in the CSF CSF: Cerebrospinal fluid.

CSF	Day 1	Day 16	Day 23	Day 30	Reference Range
Cell count (/µL)	37	94	102	67	0-5
Lymphocytes (/µL)	37	94	102	67	0-5
Proteins (mg/dL)	213	114	99	92	10-40
Glucose (mg/dL)	71	40	49	52	50-75

## Discussion

ICI therapy stimulates immune responses by reactivating cytotoxic T cells that are suppressed by tumor cells. Here, we present a rare instance of VZV meningitis in a patient undergoing ICI therapy. While encephalitis as an irAE was suspected initially, the detection of VZV DNA in the CSF confirmed VZV meningitis.

VZV infection is uncommon in patients receiving ICI [1]. A Korean cohort study indicated that patients with lung cancer undergoing ICI therapy had a lower risk of VZV infection, possibly due to the enhanced function of VZV-specific CD4+ and CD8+ T cells [2]. Recently, Morelli et al. have proposed the concept of ITI-DI as a mechanism of infection occurring in the absence of immunosuppression during ICI therapy and suggested that ICI-induced hyperinflammatory immune dysregulation may lead to the activation of infectious diseases, including VZV [3,7]. Immune checkpoints may be necessary for the establishment of latent infection and stable pathogen-host symbiosis, and their inhibition with ICIs may favor reactivation of chronic latent infection. CTLA-4 is an immune checkpoint receptor expressed on T cells, which functions as an “off switch” to regulate and suppress immune responses by outcompeting the co-stimulatory receptor CD28 for binding to B7 ligands on antigen-presenting cells [8,9]. CTLA-4 haploinsufficiency with autoimmune sequelae, lipopolysaccharide-responsive and beige-like anchor protein deficiency with autoantibodies, Treg defects, autoimmune infiltration, and enteropathy are human genetic disorders associated with deficient CTLA-4 expression and function [10]. In this case, combination therapy with ipilimumab and nivolumab was administered, suggesting the impact of CTLA-4 blockade. More accumulation of cases is needed to clarify the difference in the risk of infection, including VZV, between ICI monotherapy and combination therapy.

ICI therapy may also induce immune recovery, potentially leading to the reactivation of latent or chronic infections akin to IRIS. IRIS associated with VZV infection correlates with CD8+ T-cell responses in the CNS [5]. IRIS occurs when a recovering immune system mounts an exaggerated response to infectious agents. Originally observed in patients with HIV starting antiretroviral therapy, IRIS is now recognized in patients with cancer undergoing immune recovery through the use of ICIs. It involves the rapid restoration of pathogen-specific immunity, characterized by T-helper 1 activation and increased pro-inflammatory cytokine production [11]. Rapid immune enhancement in the context of ICI therapy may trigger IRIS-like responses, especially in patients with latent infections. This mechanism may explain the atypical presentation and prolonged CSF abnormalities in our case. The involvement of both innate and adaptive immunity in IRIS or ITI-DI aligns with the complex immunomodulatory effects of ICIs.

The atypical clinical course observed in this case is notable for several features: ICI exposure occurred before the onset of neurological symptoms; VZV infection was confirmed; and although viral replication was resolved, CSF inflammation persisted for an extended period in the absence of ongoing immunosuppression. This constellation of findings aligns with recognized patterns of ITI-DI or IRIS involving the CNS. In particular, the persistence of inflammation following viral clearance strongly suggests that the process is immune-driven rather than purely infectious. This complex interplay between ICI therapy and viral reactivation warrants further investigation. While ICIs may enhance antiviral immunity, they may also disrupt immune surveillance. Future studies should explore whether changes in specific T-cell subsets or other immunological markers can predict viral reactivation risk in patients receiving ICI therapy.

Future research should investigate whether ICI-induced immune reconstitution shares similar mechanisms with classical IRIS, as well as the precise mechanism of ITI-DI, particularly regarding the activation status of T cells and cytokine profiles. Understanding these parallels may inform strategies for preventing and managing viral reactivation or infection in patients with cancer undergoing immunotherapy.

Rechallenging ICI therapy after a severe infection such as VZV meningitis remains controversial. Current guidelines recommend that any decision to resume ICIs must carefully weigh the risks and benefits, particularly considering the severity of the initial toxicity and the potential for recurrence, which has been documented in approximately 30% of rechallenge cases [12-14]. In cases involving serious neurological events like meningitis, guideline consensus suggests that ICI rechallenge should generally be avoided unless there is clear oncologic necessity and close multidisciplinary monitoring is available [12]. Furthermore, it should be noted that in this case, the severe infection may have represented not only a direct infectious complication but also an immune-related phenomenon, such as IRIS or ITI-DI [14,15]. These syndromes reflect complex immune dysregulation rather than traditional immunosuppression, further complicating risk assessment and management decisions regarding ICI rechallenge.

## Conclusions

We report an atypical case of VZV meningitis during ICI therapy, characterized by impaired consciousness preceding the appearance of a skin rash and prolonged CSF pleocytosis. The diagnostic workup - including the temporal relationship to ICI administration, CSF analysis, and PCR detection of VZV DNA - supports a diagnosis of viral meningitis with features suggestive of immune dysregulation. The persistence of CSF pleocytosis and the unusual clinical course raise the possibility of ITI-DI or IRIS-like mechanisms contributing to disease pathogenesis. This case underscores the importance of early diagnostic testing - including polymerase chain reaction for viral pathogens - in ICI-treated patients presenting with neurological symptoms as well as the need to consider both antimicrobial therapy and immune-related processes in management. Further research is needed to clarify the mechanisms and optimize treatment strategies for similar cases at the intersection of immunotherapy and infection.
